# Supplementary material for: Light- and Melanin Nanoparticle-Induced Cytotoxicity in Metastatic Cancer Cells
Source: Pharmaceutics. 2021 Jun 26;13(7):965. doi: 10.3390/pharmaceutics13070965 (PMC8309021; doi:10.3390/pharmaceutics13070965)
Supplement: Supplementary file 1 [file pharmaceutics-13-00965-s001.zip › Supplementary.pdf]

pharmaceutics

MDPI

Supplementary Material: Light- and Melanin Nanoparticle-Induced Cytotoxicity in Metastatic Cancer Cells

Victoria R. Gabriele, Robabeh M. Mazhabi, Natalie Alexander, Purna Mukherjee, Thomas N. Seyfried, Njemuwa Nwaji, Eser M. Akinoglu, Andrzej Mackiewicz, Guofu Zhou, Michael Giersig, Michael J. Naughton and Krzysztof Kempa

Citation: Gabriele, V.R.; Mazhabi, R.M.; Alexander, N.; Mukherjee, P.; Seyfried, T.N.; Nwaji, N.; Akinoglu, E.M.; Mackiewicz, A.; Zhou, G.; Giersig, M.; et al. Light- and Melanin Nanoparticle-Induced Cytotoxicity in Metastatic Cancer Cells. *Pharmaceutics* **2021**, *13*, 965. https://doi.org/10.3390/pharmaceutics13070965

Academic Editor: Hassan Bousbaa

Received: 21 May 2021
Accepted: 23 June 2021
Published: 26 June 2021

Publisher's Note: MDPI stays neutral with regard to jurisdictional claims in published maps and institutional affiliations.

Copyright: © 2021 by the authors. Licensee MDPI, Basel, Switzerland. This article is an open access article distributed under the terms and conditions of the Creative Commons Attribution (CC BY) license (http://creativecommons.org/licenses/by/4.0/).

1. MNPs and MNPs@G Cytotoxicity and Biocompatibility

Biocompatibilities and cytotoxicity of various concentrations of MNPs (145 nm) and MNPs@G (166 nm) from 140 to 2100 μg mL^{-1} were studied via cell viability and proliferation using CCK-8 assays kits by different cell lines, A375 Melanoma, Hela, as well as Hacat cells in two different growth medium; high glucose, (4500 mg L^{-1}) and low glucose (1000 mg L^{-1}). To complete this study, we used UV-Vis spectrophotometry technique to evaluate the cell viability, cell membrane damage and cell toxicity.

The KCC-8 colorimetric assay involves metabolic bioreduction of WST-8 [2-(2-methoxy-4-nitrophenyl)-3-(4-nitrophenyl)-5-(2,4disulfophenyl)-2H-tetrazolium, monosodium salt] in the presence of 1-methoxy PMS as an electron mediator, produces a water-soluble orange colored Formazan dye. The amount of produced Formazan is directly proportional to the number of living cells and can be measured by spectrophotometric method at the absorbance of 460 nm.

In this research, the cell viability was evaluated by CCK-8 assay according to the manufacturer's instructions with some modification. Regarding to the original method, the cells was treated by MNPs and MNPs@G in a specified time. Afterward, 50–100 microliter (depends on the well plate sizes) of CCK-8 solution was added to each well into the culture medium. After 3 h incubation with CCK-8 in the incubator, the colorless WST-8 tetrazolium salt turned to a strongly orange colored solution by Formazan which the absorbance was measured spectrophotometrically (PerkinElmer, MA, USA).at 450 nm.

Scheme S1 shows the working principle of cytotoxicity detection kit which is through extracellular reduction of WST-8 by NADH (nicotineamido adenine dinucleotide reduced form) or NADPH (nicotineamido adenine dinucleotide phosphate reduced form). As it can be seen in this scheme, the reaction of dehydrogenase enzymes and their substrates (lactate dehydrogenase and lactic acid) produced in the mitochondria can generate NAD^+ or $NADP^+$ from NADH and NADPH which finally converted to a water-soluble Formazan which can dissolve directly into the culture medium. Accordingly, West 8 reaction to Formazan occurs by using ''succinate-tetrazolium reductase'' system, belongs to the mitochondrial respiratory chain which is active only in viable cells. Therefore, the tetrazolium salt is utilized as an indicator of plasma membrane damage or cell toxicity after MNP and MNP@G treatment. Therefore, in comparison with formazan concentration in well plates associates with untreated cells called as reference cells, each decrease of Formazan concentration in every well plate, would be known as the sign of cell death and the produced Formazan in the cell culture medium is in a good proportional to the concentration of applied nanoparticles.

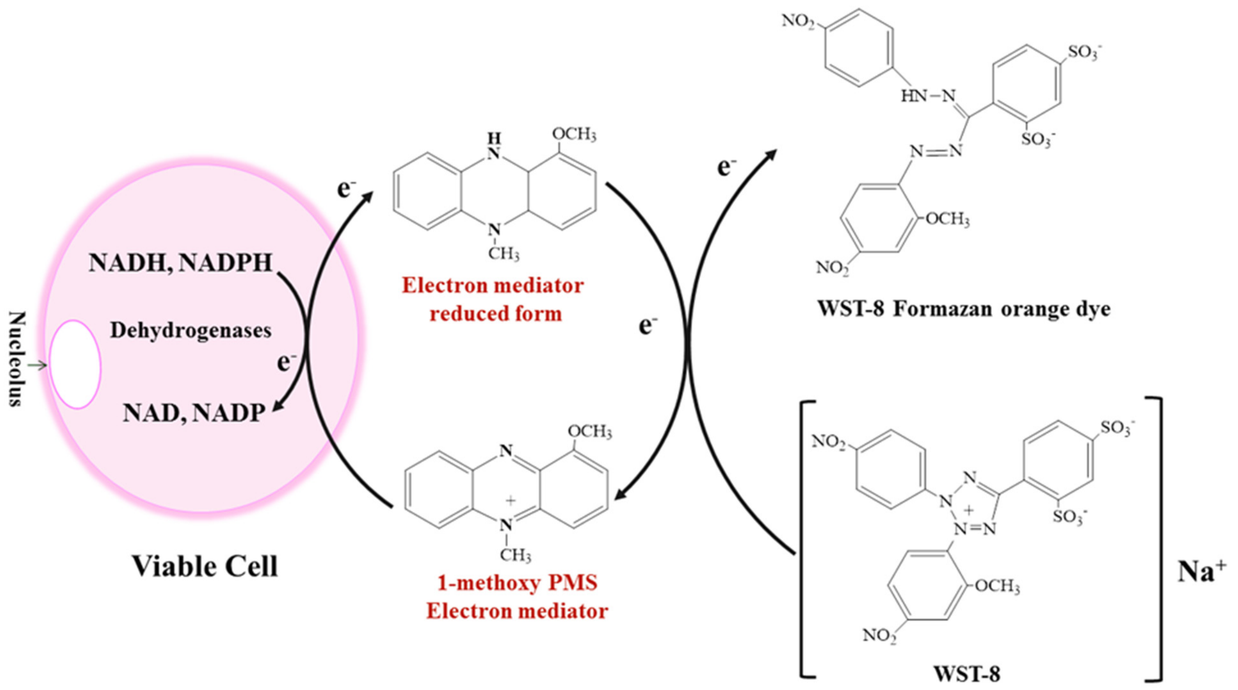

Scheme S1. WST-8 based cytotoxicity assay mechanism.

2. Comparison of Melanoma Up-Taking Quantity of MNPs and MNPs@G in Low Glucose Condition

To start, melanoma cells were seeded with density of 5×10^5 cells per well experiment in 6-well plates, in high glucose DMEM and were incubated overnight in 5% CO_2 atmosphere at 37 °C to allow adherence to the plate. The adherent cells were incubated with MNPs and MNPs@G (1400 and 3500 μg ml^{-1} in 1 microliter low glucose DMEM) for 20 hours. Afterward, 100 microliter CCK-8 (10%) were added to every well and the plate was return to the incubator.

After 3 h, the upper layer of cells was separated into a centrifuge tubes and centrifuged for 15 minutes at 1000 rpm to remove the suspended MNPs@G from culture medium. Thereafter, the UV-Vis absorption measurement were performed.

3. Culture Medium Glucose Concentration Effect on MNPs@G Cytotoxicity

To study the effect of glucose concentration on MNPs@G toxicity on melanoma cells, first, Melanoma Cells were seeded at high glucose growth medium with density of 1×10^4 cells per well in two 24-well plates. Melanoma cells were incubated overnight in 5% CO_2 atmosphere at 37 °C to allow adherence to the plate. Afterward, Melanoma cells were washed with PBS (pH 7.4). 500 μL growth medium from different glucose concentration; high glucose concentration (4500 mg L^{-1}) and low glucose concentration (1000 mg L^{-1}), were individually mixed with different concentration of MNPs@G to obtain the concentration of 0, 140, 420, 700, 980, 1400 and 2100 μg ml^{-1}. Afterward, the mixture were injected to every well containing the cells and then the cells were returned to the incubator for 72 hours incubation time for high glucose well plate and 15 h for low glucose well plate. To assess cell viability after the defined time, 50 μl of CCK-8 prepackaged solution was added to every well and the inoculated cells returned to incubator for 3 hours. The supernatant containing formazan and MNPs@G were collected, centrifuged and after removing the MNPs@G from the supernatant the absorptions were measured from 350 to 550 and the results were evaluated at maximum absorption 450 nm.

As it can be seen in figure 5 (A), the concentration of glucose has a big influence on toxic effect of MNPs@G on melanoma cell lines. In the high concentration of glucose, after 72 hours incubation with 140 μg/mL of MNPs@G, no cytotoxicity was observed while after incubation with 420 μg/ml MNPs@G at the same time, the melanoma viability decreased

to 92.6%. In the same condition, in low glucose concentration after 15 hours incubation with 140 and 420 μg mL^{-1} of MNPs@G, the viability of melanoma cancer cells were decreased to 70.2% and 40.8%. It is noticeable, in low glucose DMEM after applying 2100 μg/ml of MNPs@G, the cell viability dropped to 12.9 % even after 15 hours incubation time while in the same concentration of MNPs@G, 30.3% of melanoma cells were still alive even after 72 hours incubation in high glucose DMEM. Accordingly, as seen from figure 5 (A), to compare with high glucose, low Glucose condition extremely enhances the Cytotoxicity of MNPs@G for melanoma cell lines.

To confirm the results related to glucose concentration of DMEM on cytotoxicity performance of MNPs@G, another experiment was carried out with Hela cell line. As it can be seen from figure 5 (B), in high glucose DMEM, after 62 hours incubation with 700 μg ml^{-1} MNPs@G, the cell viability remained more than 94.8 % while at the same concentration in low glucose DMEM, the Hela cell viability dropped to 54.5 %, showing the cytotoxicity enhancing effect of MNPs@G at low glucose condition. The notable point is, by increasing the MNPs@G concentration to 2100 μg ml^{-1} after 15 hours incubation at low glucose DMEM the Hela cells viability will drops to 15.5 % while after 62 hours incubation at high glucose DMEM, 60.8% of Hela cells were still alive.

4. Control Experiment with Formazan

To do this experiment, Formazan dye was provided by applying 100 μL CCK-8 to a HeLa cell plate and was placed in the incubator. After several hours incubation, the produced formazan was collected and diluted to a certain concentration and 700 μL of this solution was added to 5 tubes containing different concentrations of mNP@G: 0, 500, 1,000, 1,500, 2,000, and 3,000 μg mL^{-1}. After 6 h mixing of mNP@G with formazan, the tubes were centrifuged at 1,000 rpm for 15 min and then mNP@G were separated from formazan solution in each tube. Finally, the absorption of the formazan solutions were measured separately by UV-Vis.

3. Zeta Potential Measurements

To confirm the above results, the zeta potential (ζ) measurements were performed using a Brookhaven NanoBrook Omni instrument in pH 7.4 HEPES buffer, with ionic strength 40 mM for mNPs, mNP@G melanoma, HeLa and HaCaT cells, with results summarized in Table 1. ζ of mNPs and mNP@G indicate good dispersion stability. The fact that ζ of the A375 cells is more negative than that for HeLa and HaCaT cells is expected, and related to surface electro-kinetic properties of the transformed cancer cells.[1]

Table S1. Zeta potential (ζ) of human cancer cells and nanoparticles in HEPES buffer, pH 7.4 with ionic strength of 40 mM. Data were obtained from 9 runs of 3 independent measurements. Right column shows the mean values and standard deviations.

Cells & Nanoparticles	ζ (mV)
melanoma	-25.5 ± 1.8
HeLa	-20.9 ± 1.0
human epidermal keratinocyte (HaCaT)	-16.9 ± 0.6
melanin nanoParticles (mNPs)	-37.2 ± 2.4
melanin nanoparticles coated with glucose (mNP@G)	-41.9 ± 0.3

Reference

1. Zhang, Y.; Yang, M.; Portney, N.G.; Cui, D.; Budak, G.; Ozbay, E.; Ozkan, M.; Ozkan, C.S. Zeta potential: a surface electrical characteristic to probe the interaction of nanoparticles with normal and cancer human breast epithelial cells. *Biomed. Microdevices* **2008**, *10*, 321–328, doi:10.1007/s10544-007-9139-2.
